# Evaluation of Resection Margins in Breast Conservation Therapy: The Pathology Perspective—Past, Present, and Future

**DOI:** 10.1155/2012/180259

**Published:** 2012-11-19

**Authors:** Rajyasree Emmadi, Elizabeth L. Wiley

**Affiliations:** Department of Pathology, University of Illinois Hospital and Health Sciences System, 840 South Wood Street, M/C 847, Chicago, IL 60612, USA

## Abstract

Tumor surgical resection margin status is important for any malignant lesion. When this occurs in conjunction with efforts to preserve or conserve the afflicted organ, these margins become extremely important. With the demonstration of no difference in overall survival between mastectomy versus lumpectomy and radiation for breast carcinoma, there is a definite trend toward smaller resections combined with radiation, constituting “breast-conserving therapy.” Tumor-free margins are therefore key to the success of this treatment protocol. We discuss the various aspects of margin status in this setting, from a pathology perspective, incorporating the past and current practices with a brief glimpse of emerging future techniques.

## 1. Introduction

The B04 study of the National Surgical Adjuvant Bowel and Breast Project (NSABP) has continued to demonstrate no significant differences in long-term survival between patients undergoing mastectomy versus lumpectomy with radiation therapy [1, 2]. The following NSABP B06 trial, a randomized prospective analysis of 1851 women, showed in a 20-year follow up that there was a cumulative incidence of 39.2% for ipsilateral tumor recurrence with lumpectomy alone and 14.3% recurrence in patients who underwent lumpectomy followed by radiation (*P* < 0.001) [1]. However it did not demonstrate a significant difference in distant-disease-free survival between the patients in the two lumpectomy groups who had tumor-free margins. Subsequently, the 10 year results of the European Organization for Research and Treatment of Cancer (EORTC) 1080 and the EORTC boost trial showed a 15% cumulative risk of local recurrence with incomplete resection margins compared to only 8% cumulative risk with microscopically tumor-free resection margins [3, 4].

Although pathologic assessment of margins for tumor is standard practice in evaluation of lumpectomies and mastectomy specimens, the obstacles for obtaining consistently accurate results are the very nature of the tissue (adiposity), the extent of in situ component [5, 6], and the insidious manner of tumor infiltration and tumor multifocality. The evaluation of surgical resection margins in any cancer surgery is important, but it becomes particularly so when considering conservation of the afflicted organ. It is therefore important to have a clear understanding of what constitutes a positive margin, the impact of disease factors in margin assessment and the different methods used to assess margins.

In this review of margin assessment, we will describe the various methods and settings in which margin assessment is performed and the advantages and disadvantages of each. We will also discuss some of methodologies employed to better predict which patients have higher risk of residual disease and shortened disease-free intervals.

## 2. Definition of Positive and Negative Margins and Tumor Clearance 

**Figure 1:**
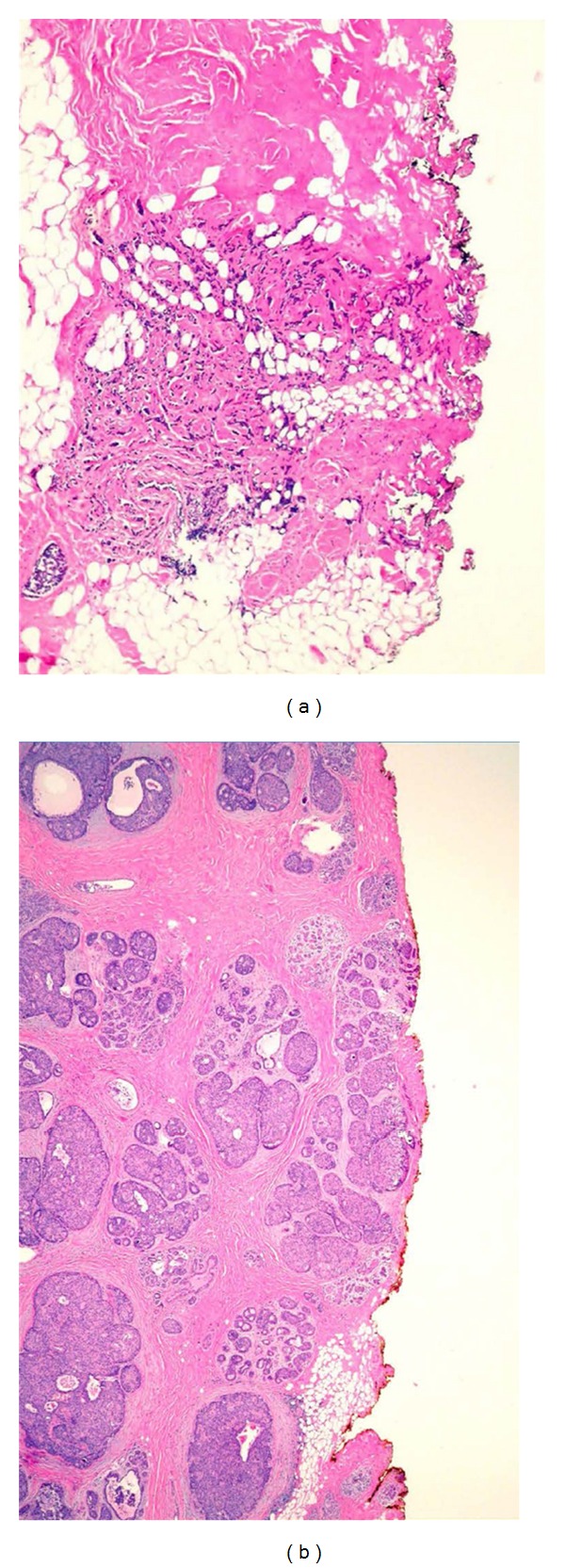
(a)   Section of an invasive carcinoma that extends to and is transected in the surgical margin. (b) Section on extensive ductal carcinoma in situ focally transected in a surgical margin. (H and E).

**Figure 2:**
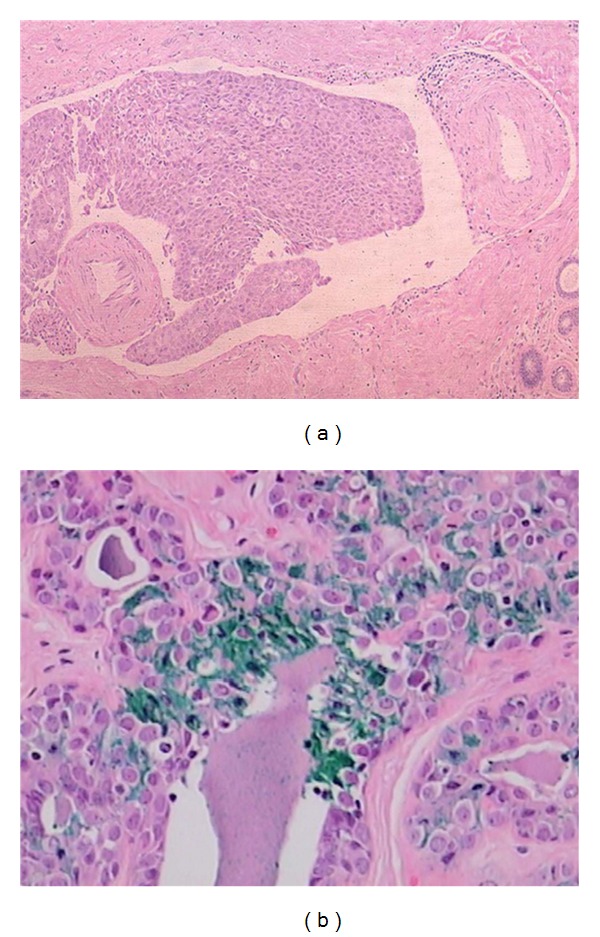
(a)  Lymphatic invasion in a margin is not considered a “positive” margin. However, such disease present in a margin indicates the patient has high risk of both residual and systemic disease. (b) Image of lobular carcinoma in situ (LCIS) in an inked margin; however, the surgical margin is not defined as being positive for carcinoma.

Classically, a margin was considered to be “positive” if invasive tumor had been cut across by the surgical blade, but margins in which tumor was close but not transected were considered “negative for tumor” (National Surgical Adjuvant Breast and Bowel Project (NSABP) B-06 study. Currently a positive margin is generally interpreted to mean the presence of tumor, either invasive and/or ductal carcinoma in situ (DCIS), at the surgical resection line (Figure 1). However, lymphatic invasion at a margin is not considered a positive margin. Neither atypical ductal hyperplasia nor lobular carcinoma in situ at margin is considered a positive margin (Figure 2).

**Figure 3:**
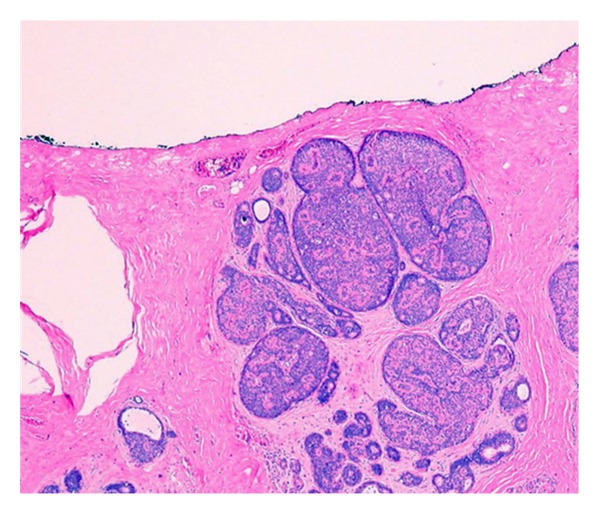
Section of ductal carcinoma in situ close to a margin but not surgically transected. There is some agreement that a clearance less than 2 mm is inadequate and places a patient at high risk for residual disease. (H and E).

What constitutes adequate clearance of tumor at the surgical margin? (Figure 3). Measurements ranging from 1–3 mm have been described as “close”. In the case of a pectoralis fascia margin, a single collagen strand separating tumor from margin is considered adequate clearance. Incised mammary tissue is considered differently. Oncoplastic surgery (combination of plastic surgery with breast-conserving treatment) defines a negative margin quantitatively as “no tumor cells within 1 cm of the cut edge of the specimen” [7] while the majority of the general literature appears to consider 2 mm as the cutoff point for a negative margin with anything less than that being considered a close margin [8]. 

 Skripenova and Layfield found residual invasive carcinoma in greater than 25% of patients with margins less than 2 mm while only 16% had residual invasive carcinoma when the margin was greater than 2 mm [9]. The incidence of invasive residual disease is also impacted by the interval between primary and secondary excision. We found a 40% incidence of residual invasive carcinoma if the secondary excision was performed within 2 weeks of the primary but less than 25% of patients had residual invasive disease when the secondary excision was performed beyond four weeks whereas the incidence of residual DCIS is not so affected [10]. 

Understandably, size, location, grade, and cosmesis all factor into the surgeon's decision of what constitutes an adequate clearance in any given patient. A survey of radiation oncologists in the U.S. and Europe shows a significant variation in the definition of a negative margin with European radiation oncologists seeming to prefer a larger tumor-free margin (>5 mm) than their American counterparts [11]. Finally, the setting in which a patient's surgery is performed and how margins are procured impact the methods of margin assessment utilized.

## 3. Tumor Characteristics and Impact on Margin Clearance

The type of tumor transected or near the resection margin is significant in terms of residual disease (RD) found on reexcision [10]. Invasive carcinoma has a lower rate of RD than DCIS near a margin. We have previously shown that this may be a consequence of greater susceptibility of invasive carcinoma to host response to injury, due to its intrinsic lack of a well-developed vascular arcade and lack of protective basement membrane and stroma when compared to DCIS [6]. 

Studies have consistently shown that patients with extensive DCIS in the primary excision are at significantly higher risk for residual tumor than those without such extensive DCIS [5, 6, 12–14]. In a more recent analysis, Dzierzanowski et al, found that the presence of DCIS in the initial core biopsy correlated with the presence of extensive DCIS (eDCIS) in the resection specimen as compared to the cores with invasive carcinoma without DCIS in the core biopsy (*P* < 0.0001). They also found a higher incidence of positive margins on lumpectomy in patients with eDCIS (38%, *P* = 0.05) [15]. 

Rodriguez et al. defined extensive DCIS as DCIS having 1 or more dimensions measuring greater than 10 mm [6]. The presence of DCIS near a margin (less than 1 mm) carries a significant risk of residual disease (50%) [6]. Schnitt et al. [5] have further shown that the presence or absence of extensive DCIS in the primary excision was of greater value in predicting the nature and extent of residual disease in the reexcision than the presence of a positive margin in the primary excision. This, however, is not the case with classic lobular carcinoma in situ (LCIS) whose presence at or near the margin is not associated with an increase in local recurrence [16].

A few groups have also shown an association between high nuclear grade or histologic subtype of DCIS and the presence of residual disease [17–19]. Sahoo et al. showed that in addition to a positive margin status, a high nuclear grade was independently associated with local recurrence. In their analysis, young age at diagnosis was also an independent predictor of recurrence. Chagpar's group found positive tumor margins to correlate with larger tumor size and the lobular subtype of breast carcinoma. Schwartz's group found micropapillary to be associated with multicentricity (86%) and comedocarcinoma more likely to be associated with microinvasive DCIS (53%). Thus, for ductal carcinoma in situ, as much as one centimeter may be needed to adequately clear disease [20].

## 4. Effect of Different Methods of Margin Procurement

The effectiveness of pathologic margin assessment is impacted by utilization of imaging and other techniques in determining the extent of breast surgery. In the case of a palpable mass, one line of resection may be followed if done using palpation guidance and another if radiologic or ultrasound imaging is employed to assess the mass and surrounding tissue. Pre- or intraoperative detection of abnormal calcifications and tumor extension will alter the final excision margin to encompass more disease and reduce the risk of positive final margins and inadequate clearance of disease [21]. 

Once a lesion of interest is excised, intraoperative palpation assessment with additional tissue taken from suspicious areas of the wall of the resection cavity, either using palpation or ultrasound guidance, can yield additional disease. Guidroz reported that surgeon assessment of the lumpectomy cavity with selective excision of additional tissue resulted in decreased need for second surgery following primary lumpectomy [22]. Simply employing a systematic removal of six additional shave margins (covering the entire cavity) from lumpectomy cavities halved the incidence of residual disease compared to patients who did not have the additional tissue submitted [23]. 

## 5. Methods of Pathologic Margin Assessment 

### 5.1. Gross Examination

**Figure 4:**
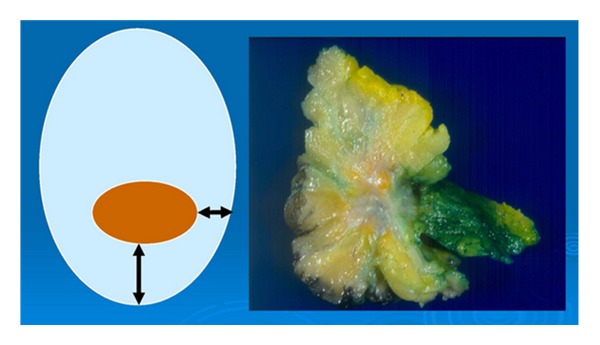
On left, gross inspection in theory: A clearly defined mass measurable from the margins. On right: Reality, an ill-defined mass with indistinct borders and irregular specimen edges.

 In theory, a mass is clearly identifiable and the distance to various resection margins measurable. In reality, often the mass is irregular with ill-defined tentacles cast out in different directions (Figure 4). An advantage of gross examination is that it is a rapid method of assessing margins and is useful in identifying grossly transected tumor and close invasive tumor. In the setting of intraoperative consultation, a grossly close or positive margin can be rapidly communicated to the operating room while additional margin assessments are completed. A grossly negative margin has little predictive value unless the margin clearance is several centimeters and the patient does not have extensive DCIS, multifocal disease or invasive lobular carcinoma. 

### 5.2. Image or Faxitron Analysis

**Figure 5:**
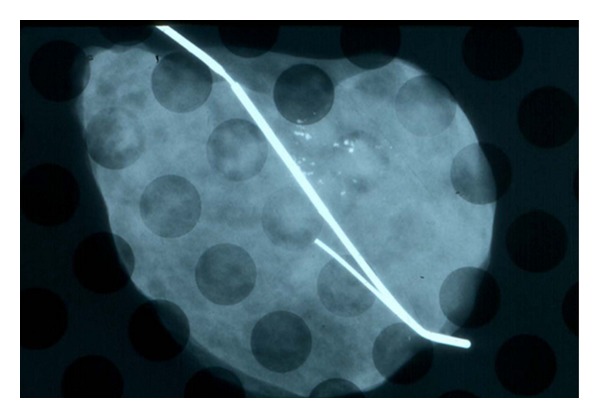
Specimen radiograph of a wire localization excission taken without compression. The cluster of abnormal calcifications is present but is not at the edge of the specimen. Often a second image is taken after rotating the specimen 90 degrees.

**Figure 6:**
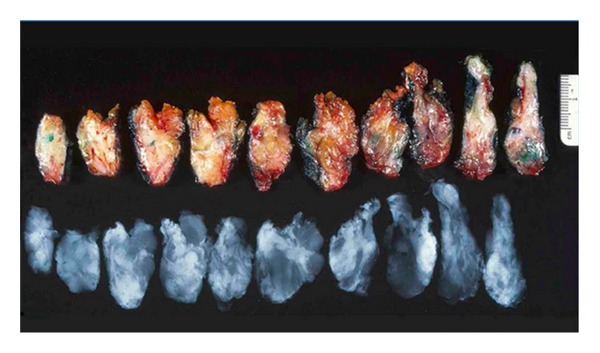
Serially sectioned excision specimen and its Faxitron X-ray. The X-ray image shows a stellate mass in the sixth section from the left with fingers extending very close to the surgical margins. (Image courtesy Dr. A. Sahin, MD Anderson Medical Center).

 Many institutions confirm resection of lesions using specimen imaging. Conventionally this is a single dimension X-ray with compression of the excision specimen (Figure 5). A smaller set of institutions incorporate 2-dimensional digital specimen mammography (Faxitron) without specimen compression. This may be followed with a second specimen mammogram of the serially sectioned specimen (Figure 6). The Faxitron appears to be better than conventional radiography at delineating microcalcifications and parenchymal distortions near margins, thereby enabling pathologists to select tissue for microscopic assessment. In the setting of intraoperative consultation, immediate reexcisions can be performed resulting in tumor-free resection margins at the time of the primary surgery [24, 25]. The sensitivity of the Faxitron appears to range from 78.6–85.6% for magnification of 1.0–2.0 : 1.0, with a specificity of 100% [26]. However, if the Faxitron equipment and the ability to interpret the images is not housed within the pathology suite, there can be significant time delay with its use in intraoperative consultation.

### 5.3. Touch Imprints or Smears of Margins

**Figure 7:**
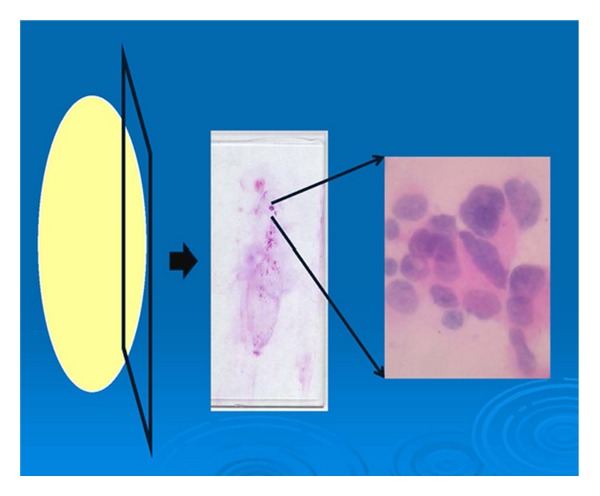
Method of obtaining touch imprints and/or smears. A slide is pressed against the surface of an excision specimen or the surface is scraped and smeared on a slide. The slide is then stained and examined for malignant cells. The microscopic image at the right shows enlarged irregular nuclei, consistent with carcinoma.

 An imprint (touch) or a scraping of the specimen surface, placed on glass slides and stained using either hematoxylin and eosin or diffquick, can be used to evaluate for tumor cells in a specimen margin (Figure 7). This method is employed only in the setting of intraoperative consultation. The advantage of this method lies in the fact that it does not alter the specimen, which can be later imaged, fixed, and/or sectioned. The disadvantages are many: the requirement for multiple imprints, the associated time consumption, the dependency on close visual inspection of the specimen, the ability to only detect transected disease, lower sensitivity, and the inability to measure the width of clearance. Klimberg et al. originally reported a sensitivity, and specificity of 100% for the use of touch preparation cytology in the evaluation of surgical margins in breast cancer [27]. The sensitivity and specificity of the method in reexcision margin assessment, however, is reportedly only 75% and 82.8%, respectively, producing a PPV of 21.4% and a NPV of 98.2% [28]. 

### 5.4. Intraoperative Frozen Section (FS)

**Figure 8:**
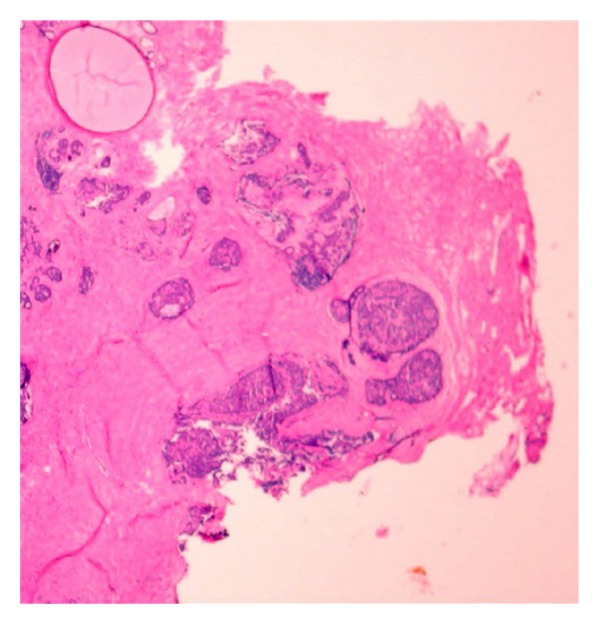
Frozen section slide showing thermal artifact, which obliterates microscopic details that a pathologist needs to diagnose carcinoma. The area bottom center on the edge of the tissue is ductal carcinoma in situ that has been transected in a margin.

 Mammary tissue is notoriously technically difficult to cryosection because of its adiposity. Freezing also introduces tissue artifact in the form of architectural distortion and resistance of adipose tissue to sectioning. In addition, if the tissue submitted for evaluation is more than one centimeter in largest dimension, there is the added risk of sampling error. This method therefore is not popular amongst most pathologists. Surgeons, however, like the method because it enables rapid microscopic examination of tissue during surgery and it can be used to determine the extent of surgery to be performed in a single operative setting. However, the use of frozen section for multiple margin assessment is time consumptive and adds significantly to operating time. In order to provide good turnaround for multiple margin assessments, a pathology frozen section suite would have to be equipped with multiple cryosectioning units and have reserves in both equipment and personnel so as not to impact other surgeries. More importantly frozen section alters the appearance of tumors, particularly ductal carcinoma in situ and infiltrating lobular carcinoma and benign lesions such as intraductal papillomas and sclerosing adenosis (Figure 8). The ability to read through the artifact and not call a benign lesion malignant or a malignant area benign is dependent on the skill and experience of both the pathologist and the entire frozen section staff. 

Cendan and his group performed a retrospective analysis of FS margin accuracy compared to permanent sections and showed an 84% concordance per case, with 24% of the patients requiring immediate reexcision intraoperatively of the lesion and approximately 20% of patients needing second surgery due to false negative margins. Expectedly, invasive lobular carcinoma and DCIS cases had higher rates of false negative FS margins. In addition, 51.2% of all patients with positive margins had at least one false-negative margin on either the primary or secondary excision [26]. 

Osborn et al. compared the cost-effectiveness of routine FS analysis of breast margins against reoperation for positive margins assessed by routine examination of the resected specimen. Their experience has shown that the use of FS for margin assessment with the attendant increased operative time provide cost savings only when the reexcision rates are greater than 36% [29]. The use of intraoperative assessment of margins is driven in part by patient demographics. Institutions which have a large patient population that travels long distances for surgical treatment will spend more resources in attempting to achieve tumor-free margins at primary excision to avoid second surgeries than medical centers whose patients are local and who can readily return for a second procedure if needed. The expense and inconvenience of patients having to return from great distances is balanced against the greater expenditure of operating room time.

### 5.5. Shave Margins

 Surgically, a shave margin is a thin piece of tissue obtained by shaving the surface of a lumpectomy cavity or other excision surface. This tissue will have two surfaces of interest: the original margin and the new margin surface. These two surfaces are differentially inked to maintain identification of the two margins. Most shave margins are large enough to require serial sectioning with submission of multiple tissue sections for microscopy to completely assess for presence or absence of disease. The pathologist can trace disease, if present, from the original margin to the new margin. Any disease present can be measured for distance from the “final” margin. 

**Figure 9:**
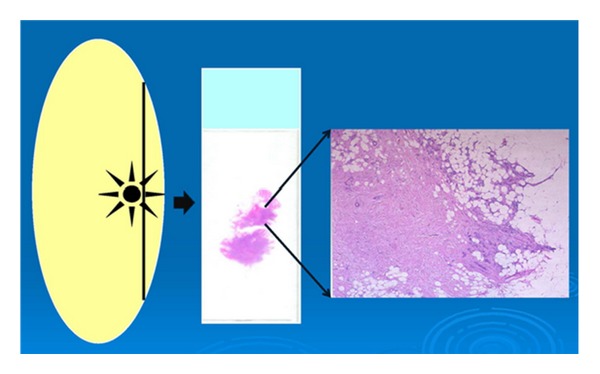
Method of obtaining a (pathologic) shave margin from a specimen. A thin piece is taken from the surface of a specimen and either frozen or processed for microscopy. The slide and microscopy show tumor present in the tissue. This would be considered a “positive” margin.

A shave margin taken by a pathologist is a very thin slice of tissue from a margin surface in question and is usually a size that can be frozen for microscopic intraoperative examination or placed directly in a tissue-processing cassette. Any tumor present in the section examined would indicate a positive margin (Figure 9). In the intraoperative setting, relatively larger surface areas can thus be examined compared to that of a perpendicular section through a margin, providing a yes or no answer. Disadvantages of a shave margin include difficulty in obtaining a shave of a soft surface and in maintaining tissue orientation. The nature of the section also precludes measurement of the clearance of a tumor from a margin. Pathologic shave margins for permanent section are most commonly used to assess margins that are distant from a tumor and are required for completeness of reporting margin status.

### 5.6. Perpendicular Margins

**Figure 10:**
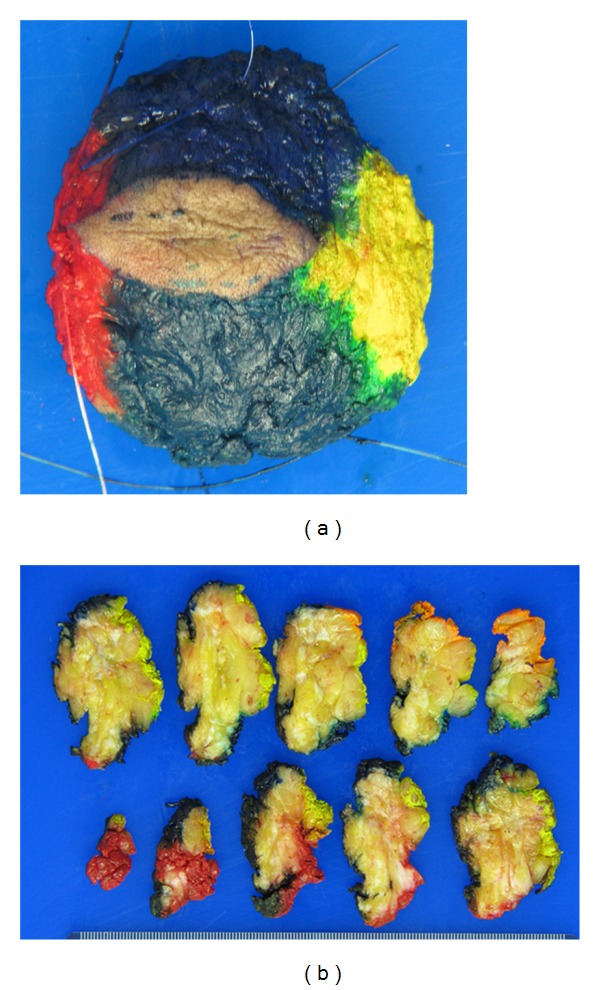
(a) Different colored inks placed on the surface of a specimen maintain the orientation during sectioning and processing. (b) Serial sectioning of inked specimen showing the different inks on the edges of the slices.

**Figure 11:**
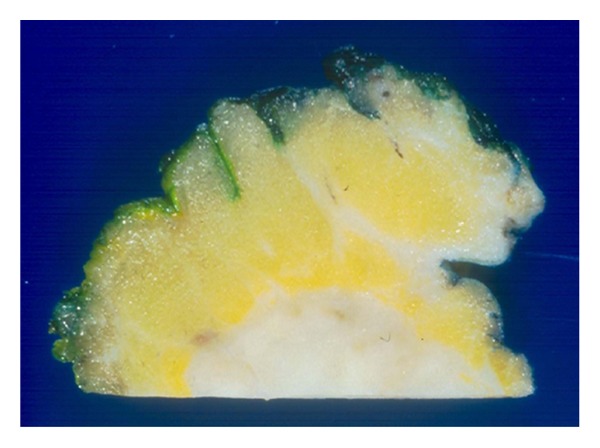
Close up of a perpendicular section of margins with tumor. The tumor is distant from the margin at the top of the image, but very close to a margin at the bottom right of the image.

 A perpendicular margin is a tissue section taken perpendicular to the margin surface. This type of margin section allows a pathologist to not only determine if a margin is positive or negative, but more importantly measure clearance of tumor from the margin. An excision specimen will be inked, either a single color or in multiple colors if the specimen is oriented and serially sectioned perpendicular to the longest axis of the tissue (Figure 10). This readily allows measurement of margin clearance both grossly and microscopically and relationship of the tumor to various margins (Figure 11). The drawback is that only a representational surface area can be examined from each section. Also, large, soft specimens are not as amenable to production of serial thin intact sections. This drawback can be mitigated by fixing a specimen utilizing special fixative with hardening agents, and/or chilling a specimen prior to sectioning. This methodology is preferred by pathologists because it allows assessment of clearance as well as tumor size, both very important factors in predicting residual disease and recurrence.

In the practice setting, pathologists will employ combinations of the above techniques to provide greater accuracy in determining margin status and the risk of residual disease being left in a patient. Good communication with the surgeon concerning how s/he is excising a lesion, whether there is additional tissue submitted separately for “margins” and the size and type of carcinoma are key. Meticulous gross examination and/or image assessment of the tissue will discover areas suspicious for tumor involvement. Such areas will be the focus of microscopic examination, both in the frozen section suite and at microscopic “sign out” of the specimen. The pathologist's goal in margin assessment is to provide an accurate assessment of margin status and accurate estimate of the risk of residual disease in each and every patient. 

## 6. New Methods for Margin Assessment 

Alternative methodologies for margin assessment have emerged recently. 

Intraoperative Optical Coherence Tomography (OCT) is a high resolution imaging technique involving real-time exvivo microscopic images up to 2 mm beneath the tissue surface. In an initial analysis the method demonstrated a sensitivity of 100% and a specificity of 82% in evaluating disease at margins [28]. 

*MarginProbe*. Quantitative diffuse reflectance spectroscopy is used to non-destructively image entire lumpectomy margins. The multichannel probe has a sensing depth of 0.5–2.2 (45–600 nm) and demonstrates a sensitivity and specificity of 79.45% and 66.7%, respectively, in an initial study. Dune Medical Devices, Inc., the sponsor of the MarginProbe is seeking premarket approval from the FDA [30]. 

## 7. Margin Index 

Margenthaler et al. retrospectively analyzed the margin status of 475 patients who underwent BCT and proposed a margin index as a more appropriate assessment of the optimum margin. The margin index was calculated using the formula: margin index = closest margin (mm)/tumor size (mm) × 100. A receiver operator curve (ROC) was created using the derived margin index and the presence or absence of residual disease in the reexcision specimen. A margin index >5, producing a sensitivity of 85% and specificity of 73%, was found to equate with a 3.2% risk of finding residual disease [31]. 

## 8. Recommendations 

While there is consensus on what constitutes a positive margin, there is still no consensus on what constitutes an adequate clearance. As neither the NSABP-B04 nor B06 trials ever defined clearance or close margin, we recommend that objective data be incorporated in routine reporting. We utilize the format of “surgical resection margins are free of tumor/negative for carcinoma”, and specifying the closest margin “with a clearance of “X” mm” in the main report. Documentation of margin clearance is also a component of the College of American Pathologists' (CAP) Breast Cancer Case Summary protocol. 

We concur with Morrow et al. that systemic chemotherapy that reduces the risk of distant metastases also likely reduces the risk of local recurrence [32]. However, we believe that there may not be one standard for clearance as tumor biology probably dictates that determination. Ultimately, objective reporting formats may provide the correlative data needed to stratify clearance requirements based on grade, receptor status, and planned systemic chemotherapy.

We therefore recommend compliance with CAP Breast Cancer summary protocols and that all mammary tumor excisions (lumpectomy, mastectomy) routinely incorporate not only the margin status (positive/negative), but also document the width of clearance at the closest margins, particularly those less than 2 mm which have been shown to carry a >25% risk of residual disease. 

## 9. Conclusion

Over the past fifty years, treatment of breast cancer has evolved from a single, radical procedure to techniques that limit the extent of surgery while improving disease free survival and overall survival of patients. With the introduction of limited surgical excision has come the need for accurate assessment of excision margins both intraoperatively and postoperatively. We have defined what constitutes a positive and a negative margin and why tumor clearance rather than just a “negative” margin is important in eliminating residual disease. We have outlined the various methods of pathologic assessment of margins and the settings in which they are employed and two new techniques that have potential to provide assessment in real time.
